# Protocol for the *ADDITION-Plus *study: a randomised controlled trial of an individually-tailored behaviour change intervention among people with recently diagnosed type 2 diabetes under intensive UK general practice care

**DOI:** 10.1186/1471-2458-11-211

**Published:** 2011-04-04

**Authors:** Simon J Griffin, Rebecca K Simmons, Kate M Williams, A Toby Prevost, Wendy Hardeman, Julie Grant, Fiona Whittle, Sue Boase, Imogen Hobbis, Soren Brage, Kate Westgate, Tom Fanshawe, Stephen Sutton, Nicholas J Wareham, Ann Louise Kinmonth

**Affiliations:** 1 MRC Epidemiology Unit, Box 285, Addenbrooke's Hospital, Hills Road, Cambridge, CB2 0QQ, UK; 2 General Practice and Primary Care Research Unit, Department of Public Health and Primary Care, University of Cambridge, Robinson Way, Cambridge, CB2 0SR, UK; 3 King's College London, Department of Primary Care and Public Health Sciences, London, SE1 3QD, UK

## Abstract

**Background:**

The increasing prevalence of type 2 diabetes poses both clinical and public health challenges. Cost-effective approaches to prevent progression of the disease in primary care are needed. Evidence suggests that intensive multifactorial interventions including medication and behaviour change can significantly reduce cardiovascular morbidity and mortality among patients with established type 2 diabetes, and that patient education in self-management can improve short-term outcomes. However, existing studies cannot isolate the effects of behavioural interventions promoting self-care from other aspects of intensive primary care management. The *ADDITION*-*Plus *trial was designed to address these issues among recently diagnosed patients in primary care over one year.

**Methods/Design:**

*ADDITION-Plus *is an explanatory randomised controlled trial of a facilitator-led, theory-based behaviour change intervention tailored to individuals with recently diagnosed type 2 diabetes. 34 practices in the East Anglia region participated. 478 patients with diabetes were individually randomised to receive **(i) **intensive treatment alone (n = 239), or **(ii) **intensive treatment plus the facilitator-led individual behaviour change intervention (n = 239). Facilitators taught patients key skills to facilitate change and maintenance of key behaviours (physical activity, dietary change, medication adherence and smoking), including goal setting, action planning, self-monitoring and building habits. The intervention was delivered over one year at the participant's surgery and included a one-hour introductory meeting followed by six 30-minute meetings and four brief telephone calls. Primary endpoints are physical activity energy expenditure (assessed by individually calibrated heart rate monitoring and movement sensing), change in objectively measured dietary intake (plasma vitamin C), medication adherence (plasma drug levels), and smoking status (plasma cotinine levels) at one year. We will undertake an intention-to-treat analysis of the effect of the intervention on these measures, an assessment of cost-effectiveness, and analyse predictors of behaviour change in the cohort.

**Discussion:**

The *ADDITION-Plus *trial will establish the medium-term effectiveness and cost-effectiveness of adding an externally facilitated intervention tailored to support change in multiple behaviours among intensively-treated individuals with recently diagnosed type 2 diabetes in primary care. Results will inform policy recommendations concerning the management of patients early in the course of diabetes. Findings will also improve understanding of the factors influencing change in multiple behaviours, and their association with health outcomes.

**Trial registration:**

ISRCTN: ISRCTN99175498

## Background

Diabetes presents a global challenge to health and well being. The total number of people with the condition is projected to rise from 285 million in 2010 to 438 million in 2030 [1]. More than 2.5 million people in the UK are estimated to have diabetes, and the condition is the leading cause of kidney failure, blindness in adults, and amputations in the UK [2]. The condition can affect mental health and well-being, and is a major risk factor for heart disease and stroke, reducing life expectancy on average by 10 years [3].

The clinical care of people with diabetes currently consumes around 10% of the NHS budget, a total exceeding £9 billion [2]. People with diabetes (or their carers) are responsible for the day-to-day management of their condition, which includes eating a healthy diet, being physically active, taking medication as prescribed and not smoking. There is scope for improvement in the prevalence of health-promoting behaviours among people at risk of and with diabetes [4-6]. Developing effective approaches to prevent progression of the disease by intensification of primary care management, including prescribing and organisation of services, and by directly supporting individual patients in self care, is important in tackling the growing public health burden of type 2 diabetes [7,8].

### Evidence to support behavioural interventions to reduce cardiovascular risk

There is good evidence that the onset of diabetes can be delayed, and cardiovascular risk factors reduced, by intensive interventions targeting multiple behaviours among large groups of individuals with impaired glucose tolerance followed up over several years [9]. The lifestyle interventions in these programmes were individually tailored and delivered by case managers or nutritionists with extended training. They drew on a range of theoretical perspectives and used techniques such as goal setting and self monitoring. In these studies the risk of diabetes was directly associated with change in lifestyle: the more behavioural targets that the participants achieved at follow-up, the lower their incidence of diabetes [10,11].

It has been recognised for some time that well organised care, including regular recall and review of patients, is associated with better outcomes [12]. More recently, there is also evidence from primary care that regular patient follow-up supported by prompting of doctors, feedback on goal attainment, and continuing medical education and guidelines can improve cardiovascular risk factors in patients followed 8 years from diagnosis compared with routine care [13]. Later in the disease trajectory there is evidence from secondary care that intensive multifactorial intervention including prescription of medication and behavioural advice compared with routine care, can significantly reduce cardiovascular mortality in patients with type 2 diabetes and microalbuminuria after 8 years. The results showed a 50% reduction in cardiovascular and microvascular events in the intervention group compared with routine care. However, the intervention had only minor effects on carbohydrate and fat intake, and no significant differences were observed for self-reported smoking and exercise habits between groups [14]. The Look AHEAD (Action for Health in Diabetes) trial is a multi-centre, clinic-based randomised trial comparing the effects of an intensive lifestyle intervention with diabetes support and education (control group) on cardiovascular risk factors and major cardiovascular disease among 5,145 overweight or obese individuals with clinically-detected type 2 diabetes [15]. Four-year results from the trial are encouraging, with participants in the intensive lifestyle arm achieving greater weight loss and greater improvements in fitness, glycated haemoglobin, systolic blood pressure, and HDL-cholesterol than those in the control group. The intervention was very intensive and participants were followed up annually. Overall, the evidence to date suggests that the greatest impact on cardiovascular risk is from intensified prescribing rather than patient education or behavioural interventions [7,8]. Despite this there is widespread introduction of healthy lifestyle facilitators, or health trainers, throughout England [16].

### Limitations to the evidence to support diabetes management in primary care

While evidence from earlier studies is encouraging, it is not clear which approaches or combinations can cost-effectively support behavioural change in people with newly or recently diagnosed type 2 diabetes in primary care. Most of the studies that demonstrate benefits from interventions targeting multiple behavioural risk factors have been conducted in research clinics or specialist settings. In particular it is unclear whether direct intervention by specialist lifestyle trainers to facilitate behaviour change can add significantly to intensive management by core multidisciplinary primary care teams. In the primary care of type 2 diabetes there are now an increasing number of trials of case management, and self-management of diabetes that include a focus on key risk behaviours; lack of regular physical activity, unhealthy dietary intake and smoking. Reviews of randomised studies [17,18] support the effectiveness of patient self-management training in improving the management of type 2 diabetes in the short term. However, the contents of many interventions have not been clearly specified and measurement of quality of delivery or receipt is largely lacking. It is thus difficult to unravel why the effectiveness of behavioural interventions is so variable. With longer follow-up (>6 months), some of the behavioural interventions that used regular contact with patients for reinforcement were effective in improving glycaemic control. Educational interventions using a patient-centred approach to goal setting and problem-solving were more promising than didactic interventions in improving glycaemic control, weight, and lipid profiles [17-20]. Interventions based primarily on providing information about how to behave to avoid negative health outcomes are less effective than those using well-specified behaviour change techniques, building on participants' own motivations within a psychological framework [21,22].

Most of these studies omit the behaviour of medication taking. Yet intensive use of multiple medications to control cardiovascular risk factors has been a central tenet of effective interventions to improve cardiovascular outcomes in type 2 diabetes over the last ten years [23,24]. Patients can find taking medication regularly difficult, particularly for multiple drugs in asymptomatic conditions [25,26], emphasising the need for interventions to support medication taking as prescribed alongside other health-related behaviours.

A major limitation of studies to date is the inability to isolate the effects of behavioural interventions from other aspects of intensified management including prescribing and organisation of services. This applies to both the effect of the intervention on behaviour, and the subsequent effect of behaviour change on clinical outcomes such as cardiovascular risk. The measurement of the behaviours themselves is a challenge, with the majority of trials relying on self-report, which is imprecise and susceptible to recall bias. Few behavioural interventions specify a hypothesised mechanism of action of behaviour change techniques, and only a minority measure behaviour and its hypothesised determinants along a specified causal pathway. This has limited the ability to identify active ingredients of interventions aimed at facilitating behaviour change and maintenance [27] and to replicate effective interventions in specific clinical settings.

No studies of behavioural management of type-2 diabetes in primary care early in the trajectory of the disease have demonstrated effects on cardiovascular disease-related events or mortality; economic analyses are absent or limited; and few studies examine health-care utilization or patient-centred outcomes such as quality of life or functional status. Performance, selection, attrition, and detection biases are common in the studies reviewed, and external generalisability is often limited.

Large gaps thus remain in the evidence concerning effectiveness and cost-effectiveness of interventions to improve multiple cardiovascular risk factors among patients with type 2 diabetes in primary care by behavioural means [20,28]. It is unclear whether interventions, based on theory and evidence from psychology [29,30] and designed to facilitate and maintain changes in behaviour (physical activity, diet, medication adherence and smoking), could be cost-effective among people detected early in the trajectory of the disease. It is also unclear how such interventions are best delivered within a health service. The existing evidence points to the importance of clinical and social context of both the patient and practitioner in enabling or obstructing health-related behaviour changes, and of a coherent approach by primary and secondary care [20]. The *ADDITION-Plus *trial was established to address these gaps.

**Figure 1:**
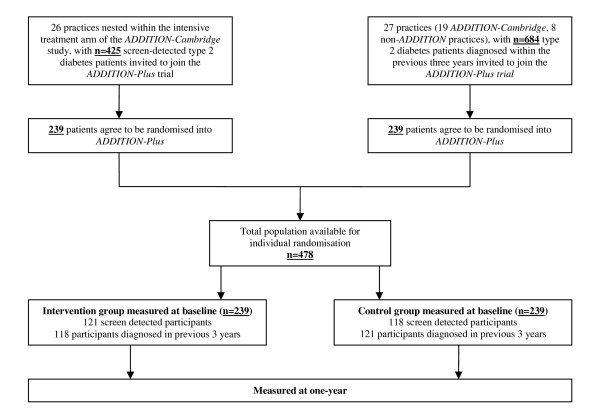
**Design and participant flows in the *ADDITION-Plus *trial (Cambridge, UK)**.

The *ADDITION-Plus *trial was originally established within the intensive treatment group in the Cambridge centre of the *ADDITION *study (Figure 1). In brief, the Anglo-Danish-Dutch Study of Intensive Treatment of people with newly diagnosed diabetes in primary care (*ADDITION*) study was set-up in 2001 [31]. It is a primary care-based study consisting of a screening phase followed by a pragmatic open-label cluster randomised controlled trial comparing the effect on cardiovascular risk of intensive multi-factorial therapy and theory-based diabetes education delivered by practice nurses (intensive treatment) with routine care in patients with screen-detected diabetes.

The aim of *ADDITION-Plus *was to assess whether a behaviour change intervention delivered by trained and quality-assured lifestyle facilitators, external to the primary care team, was a cost-effective addition to intensive treatment and could achieve and maintain changes in important health related behaviours (physical activity, dietary change, medication adherence and smoking cessation) when offered to people with recently diagnosed diabetes already receiving intensive general practice based care. Follow-up was at one year post-randomisation. The trial recruited screen-detected type 2 diabetes patients from within the *ADDITION-Cambridge *trial [32], and included patients with type 2 diabetes clinically diagnosed within the previous three years. As such, the *ADDITION-Plus *trial allows estimation of the additional contribution of a facilitator-led intervention to good practice care in the optimisation of healthy behaviours and well-being, and to the reduction in cardiovascular risk factors in individuals with recently diagnosed diabetes.

### ADDITION-Plus objectives

To quantify the effectiveness and cost-effectiveness of adding a facilitator-led theory-based behaviour change intervention to intensive treatment of screen- and clinically- detected type 2 diabetes patients, and to analyse the psychological, socio-demographic and clinical predictors of behaviour change in the cohort. The following research questions are posed:

### Trial analysis

1. Can a facilitator-led intervention, based on theory and evidence from psychology, to increase and maintain health-promoting behaviours (physical activity, dietary change, taking medication and smoking cessation) achieve clinically important and measurable change in these behaviours when offered to people with screen-detected diabetes and to those recently diagnosed with diabetes?

Primary outcomes include *objectively measured *physical activity, fruit and vegetable intake, medication adherence, and smoking status.

Secondary outcomes include (i) a composite measure of behaviour derived from the four primary outcomes; (ii) self-reported behaviours; (iii) modelled cardiovascular risk (UKPDS risk engine, v3[33]); (iv) levels of individual clinical risk factors; (v) functional status, health utility and quality of life; and (vi) costs and cost-effectiveness.

### Cohort analysis

2. What and how changes were achieved in objectively measured health behaviours and health outcomes across the trial cohort?

2.1 What are the psychological, socio-demographic and clinical predictors of self-reported and objectively measured behaviour change?

2.2 What are the clinical consequences of behaviour change?

## Methods and Design

### Design and setting

The *ADDITION-Plus *study is nested within the intensive treatment arm of the *ADDITION-Cambridge *trial with additional practices (n = 8) added to increase the recruitment of recently diagnosed patients; see Figure 1 for details of study design and patient flows. Participants were recruited from 34 general practices in urban, suburban and rural Cambridgeshire, East Hertfordshire, West Suffolk and North Essex areas of England. 239/425 eligible screen-detected patients from *ADDITION-Cambridge *study, and 239/684 patients clinically diagnosed within the previous 3 years were individually randomised to receive intensive treatment alone or in conjunction with a facilitator-led behaviour change intervention.

Ethical approval was obtained from the Eastern Multi-Centre Research Ethics Committee (reference number: 02/5/54). Participants gave written informed consent to take part in the study. ISRCTN 99175498.

### Participants

Eligibility criteria (initially assessed by general practice staff) included: age (40-69yrs) with type 2 diabetes following (i) screening in the *ADDITION *programme or (ii) clinical diagnosis during the three previous years in participating GP surgeries. Exclusion criteria included women who were pregnant or lactating or anybody who had a psychotic illness or an illness with a likely prognosis of less than one year.

Individual randomisation of participants to one of two carefully characterised interventions was independent of intervention facilitators, study coordination and measurement teams, and central using a partial minimisation procedure that dynamically adjusted the randomisation probabilities to balance stratifiers (age, sex and general practice, and within screen-detected and clinically-diagnosed subgroups: smoking, self-reported medication adherence [34] and BMI). The stratifiers were individually weighted in order that those with more categories (such as general practice) could achieve an even distribution between the two arms. A random element was included in the minimisation to allow a sound basis for inference.

### Intensive treatment (comparison arm)

The following features were added to routine multi-disciplinary primary care of diabetes to achieve intensive treatment in both trial groups:

1. Academic detailing for general practitioners and practice nurses: A practice-based academic detailing session for primary care teams was conducted by a local diabetologist or GP opinion leader to describe the treatment algorithms and targets, patient materials, and to present the evidence underpinning intensive treatment.

2. Treatment algorithms: These were based on trial data demonstrating the benefits of intensive treatment of several cardiovascular risk factors in people with diabetes [35-38], and recommended medication within licensed indications (Table 1). GPs were advised to consider prescribing an angiotensin converting enzyme (ACE) inhibitor to patients with a blood pressure≥120/80 and either a previous cardiovascular event or at least one other cardiovascular risk factor [39]. The remainder of the intervention was based on the stepwise target-led regimen from the Steno-2 study [23] aimed at optimising hyperglycaemia, hypertension, dyslipidaemia and microalbuminuria. GPs were advised to consider prescribing 75 mg of aspirin daily to patients without specific contraindications. Although targets for treatment were specified and classes of drugs recommended, where there was a choice between individual drugs the decision was made by the GPs and patients. The intensive treatment protocol was revised after publication of the Heart Protection Study [40] to include a recommendation to prescribe a statin to all patients with a cholesterol level of≥3.5 mmol/l.

**Table 1:** Measures used at baseline and one year in the *ADDITION-Plus *trial (Cambridge, UK)

Measures	Baseline		One year	
	**C**	**I**	**C**	**I**

***Objectively measured health behaviours***				

Physical activity (ActiHeart)[47]			X	X

Dietary intake (plasma vitamin C concentration)	X	X	X	X

Medication adherence (plasma drug concentrations)			X	X

Smoking status (plasma cotinine concentration)			X	X

***Self-reported health behaviours***				

Physical activity: EPAQ-2 [52], IPAQ [53]	X	X	X	X

Dietary intake: EPIC food frequency questionnaire [54]	X	X	X	X

Medication adherence:				

All drugs during the last month [34]	X	X	X	X

Hypoglycaemic drugs during the last month [34]			X	X

Smoking status and alcohol consumption (questionnaire)	X	X	X	X

***CVD risk***				

Modelled ten-year CVD risk: UKPDS risk engine, v3 [33]	X	X	X	X

Self-reported history of angina, heart attack and stroke	X	X	X	X

***Biochemical measures***				

HbA_1c_, total cholesterol, HDL and LDL cholesterol, triglyceride, urea and electrolytes, creatinine, albumin, bilirubin, alanine amino transferanse (ALT), alkaline phosphatase, aspartate aminotransference (AST), thyroid stimulating hormone (TSH), urine albumin/creatinine ratio	X	X	X	X

***Clinical measures***				

Waist circumference, height, weight, blood pressure, pulse, body fat impedance and ECG	X	X	X	X

Cardiorespiratory fitness measured using a sub-maximal VO_2 _test[47]			X	X

Rose angina questionnaire [55]			X	X

Neuropathy questionnaire (adapted from the Michigan screening instrument) ^1^	X	X	X	X

***Health utility, functional status, quality of life, well-being, treatment satisfaction and anxiety***				

Diabetes well-being (WBQ-28) [57]			X	X

SF-36 [74]			X	X

Diabetes-related quality of life (ADDQOL) [57]			X	X

Diabetes Treatment satisfaction (DTS) [57]			X	X

EuroQol EQ-5D [59]	X	X	X	X

Consultation and relational empathy measure (CARE) [61]			X	X

Spielberger Short form State anxiety inventory [60]	X	X	X	X

Assessment of diabetes services			X	X

Frequency of and views about self-monitoring of blood glucose (DiGEM questionnaire [62])			X	X

***Beliefs about behaviour change, illness perceptions and habit***				

Beliefs about behaviour change: intention, perceived behavioural control, behavioural beliefs (physical activity, eating a low-fat diet; taking medication and smoking cessation) [29]	X	X	X	X

Illness perceptions (consequences and treatment control sub-scales IPQ-R) [65]	X	X	X	X

Diabetes knowledge^1^			X	X

Habit [67]) in relation to physical activity and dietary change			X	X

***Intervention evaluation***				

Facilitator assessment				X

Skills in last 12 months in relation to lifestyle change and medication taking^1^				X

***Costs***				

Self-reported current medication/vitamins	X	X	X	X

Personal patient costs^1^	X	X		

Health service and medication use in the previous three months (adapted from the Aberdeen Health Service Research Unit questionnaire) ^1^			X	X

C = control group; I = intervention group; ^1 ^Questionnaire developed for the study

3. Treatment targets: HbA_1c _<7% (with a recommendation to initiate treatment at a threshold of 6.5%), blood pressure≤135/85 mmHg, total cholesterol <5 mmol/l or < 4.5 mmol/l for people with a history of ischaemic heart disease.

4. Audit and feedback: Interactive practice-based audit and feedback sessions were organised around 6 and 14 months after the academic detailing session and annually thereafter. They consisted of discussion of overall achievement of treatment targets and optimisation of the management of individual patients.

5. Provision of glucometers for patients: Blood glucometers and training in their use were provided to the primary care team. However, the decision to offer a glucometer to a patient was left to practitioners.

6. Theory-based diabetes education: Practice nurses were provided with theory-based education materials to discuss with, and give to patients in order to provide a shared framework for the causes, consequences and treatment of diabetes ('*Getting Started with Diabetes*'). The materials were developed by a multidisciplinary team and drew on Leventhal's Common-Sense Model [41]. They cross-referred to '*Diabetes for Beginners-Type 2' *a Diabetes UK publication [42] that was included in the patient information pack. The materials stressed the importance of a healthy lifestyle for the control of diabetes and associated health problems and provided targets for behaviour change. Specifically, participants with a BMI > 28 kg/m^2 ^were encouraged to lose 5-10% of their body weight, and all participants to increase physical activity gradually (recommendations to reach the equivalent of 35 minutes of brisk walking per day for 7 days per week), decrease the consumption of fatty foods and sugar, increase the consumption of fruit, vegetables, and whole grains, avoid excessive alcohol intake, take their medication regularly, self-monitor their blood glucose level (if applicable) and attend annual health checks. Participants who smoked were encouraged to stop.

7. Funding to support more frequent contact between patients and practitioners: Practices received funding equivalent to three 10-minute consultations with a GP and three 15-minute nurse appointments per patient per year.

8. Dietary counselling: GPs were recommended to refer all newly diagnosed patients to a dietician.

### Intervention group: Intensive treatment plus facilitator led behaviour change intervention

Participants in this group received intensive treatment (described above) plus a facilitator-led, individually tailored behaviour change intervention, based on psychological theory and evidence. The intervention was delivered by trained lifestyle facilitators, who were not part of the practice team. The facilitators used detailed protocols for each contact with the participant and received on-going supervision and feedback from a Clinical Psychologist, informed by assessment of tape-recorded consultations, enabling tight quality assurance of intervention delivery. The intervention was designed to build on the diabetes education delivered by practice nurses and intensive treatment by the practice team. The behaviours targeted in the intervention were physical activity, dietary intake, medication adherence, and smoking cessation. Hypothesised mediators of behaviour change targeted in the intervention included illness perceptions based on Leventhal's Common-Sense Model [41] and beliefs about behaviour change based on the Theory of Planned Behaviour (TPB) [29]. The intervention targeted instrumental (e.g. health) and affective beliefs about changing specific behaviours, (lack of) encouragement by important others (subjective norm), and perceived barriers and facilitators of behaviour change (perceived behavioural control). The behaviour change techniques used by the facilitators could be mapped onto the TPB (e.g., giving information, strengthening motivation), Operant Theory (identifying cues for action and reinforcement of behaviour change or effort), Carver and Scheier's Control Theory (e.g., goal setting, action planning, self-monitoring) and Relapse Prevention Theory (e.g., preparing for and dealing with setbacks) [29,43-45]. Facilitators taught patients this range of self-regulatory skills to achieve behaviour change and maintenance over time, which was supported by a manual describing the skills. Each patient initially selected to work on the behaviour(s) they most wanted to change and felt most confident about changing. The facilitator later discussed with them other domains in which change was possible and encouraged patients to consider setting additional goals. Interested patients were offered pedometers and/or glucometers. The intervention was delivered over one year at the participants' surgeries. It included a one-hour introductory meeting followed by six 30-minute meetings and four brief phone calls. Facilitators visited participant's homes or workplaces if it was not possible to meet in the surgery.

### Measurement

Table 1 shows the measures taken at each stage in the *ADDITION-Plus *study. Baseline measurements were carried out on all eligible patients including the completion of questionnaires, physiological and anthropometric measures, and venesection. Similar measurements were conducted one year after recruitment. Measurements were undertaken at outpatient clinical research facilities by trained staff following standard operational procedures and unaware of participants' study group allocation. Double data entry of all anthropometric and questionnaire measures was undertaken by experienced, independent agencies, blind to study group (Wyman Dillon Research and Data Management, Bristol, UK and Document Technologies and Imaging Solutions Ltd, Chalgrove, Oxford, UK).

#### Objectively measured health behaviours

Physical activity was assessed at one-year using a combined heart rate and movement sensor (Actiheart, CamNtech, Cambridge, UK), which was worn continuously for at least 4 days [46]. A graded treadmill walk test was used for individual calibration of heart rate [47], a procedure which also provides an estimate of cardio-respiratory fitness by extrapolation of the HR-VO2 relationship to age-predicted maximal heart rate [48]. Heart rate data collected during the free-living period were pre-processed [49] and activity intensity (J/min/kg) was estimated using a branched equation framework [47,50]. Resulting time-series data were summarised into physical activity energy expenditure (PAEE, in kJ/kg/day) and time spent in sedentary and moderate-to-vigorous intensity physical activity (SPA and MVPA, min/day), whilst minimising diurnal information bias caused by non-wear periods (segments of non-physiological data). Participants without individual calibration data but who provided data during free-living were processed using an age, sex, beta blocker, and sleeping heart rate adjusted group calibration equation for the translation of heart rate into activity intensity.

Dietary intake was assessed at baseline and one-year by plasma vitamin C levels. Vitamin C is not endogenously produced and therefore provides a robust measurement of consumption. Medication adherence was assessed at one-year by plasma drug levels. Concentrations of metformin, simvastatin and atorvastatin in plasma samples were measured by liquid-chromatography mass-spectrometry (LC-MS/MS) after protein precipitation extraction. The LC-MS/MS methods for simvastatin and atorvastatin were established at Quotient Bioresearch Ltd. (Fordham) for the *ADDITION-Plus *study. The metformin method was validated at Quotient Bioresearch Ltd. (Fordham) for a previous study [51]. The calibration ranges for each of the analytes were as follows: metformin 5.00-5000 ng/mL, simvastatin 0.100-50.0 ng/mL and atorvastatin 0.0600 to 30.0 ng/mL. Objective measurement of smoking was assessed by analysis of cotinine levels in plasma samples using an Immulite^® ^Nicotine metabolite solid phase competitive chemiluminescent immunoassay (Siemens).

#### Self-reported health behaviours

Physical activity was assessed using the validated EPAQ2 [52] and IPAQ [53] questionnaires. Dietary intake was evaluated using a validated food frequency questionnaire [54]. Medication adherence was assessed by the Medication Adherence Report Schedule (MARS) questionnaire [34]. Smoking status and alcohol consumption were assessed by questionnaire.

#### Modelled CVD risk

The UKPDS model [33] uses information on sex, ethnicity, smoking status, presence or absence of atrial fibrillation, systolic blood pressure, HbA_1c_, total cholesterol, and HDL-cholesterol to predict the 10-year risk of primary CVD. Predicted events include myocardial infarction, sudden cardiac death, other incident ischaemic heart disease, stroke, and peripheral vascular disease death.

#### Biochemical measures

HbA_1c _was analysed in venous samples at the time of diagnostic testing by ion-exchange high-performance liquid chromatography on a Tosoh machines (Tosoh Bioscience, Redditch, UK). Serum total cholesterol, HDL-cholesterol and triglycerides was measured by means of enzymatic techniques (Dade Behring Dimension analyser, Newark, USA). Plasma creatinine was analysed with kinetic colorimetric methods, and urine albumin by rate nephelemetry (Dade Behring Nephelometer II, Newark, USA). Plasma levels of urea and electrolytes, bilirubin, alanine aminotansferase (ALT), aspartate aminotransferase (AST), alkaline phosphatase, and thyroid stimulating hormone (TSH) and urine levels of creatinine were assayed by means of the Dade Behring Dimension analyser. Metaphosphoric acid was added to plasma samples for vitamin C analysis before being stored at -80C°. Plasma vitamin C level was measured with a Fluoroskan Ascent FL fluorometer. The albumin-to-creatinine ratio (ACR) was measured on a random spot urine specimen.

#### Clinical measures

Blood pressure was calculated as the mean of three measurements performed after at least 10 minutes rest, while participants were seated with the cuff on the predominant arm at the level of the heart, using an automatic sphygmomanometer (Omron M4, UK). ECG was recorded by a 12 lead machine. Body height and weight were measured in light indoor clothing and without shoes using a fixed rigid stadiometer and a scale (SECA, UK) respectively. Waist circumference was estimated as the average of two measurements taken with a tape measure halfway between the lowest point of the rib cage and the anterior superior iliac crests when standing. Body fat percentage was measured by bio-electrical impedance (TANITA, Tokyo, Japan). Angina was assessed using the Rose angina questionnaire [55]. Neuropathy was evaluated using an adapted version of the Michigan Neuropathy Screening Instrument [56].

#### Health utility, functional status, quality of life, well-being, treatment satisfaction and anxiety

The generic and disease-specific instruments used were diabetes well-being questionnaire (W-BQ12) [57], SF-36 [58], Audit of Diabetes-Dependent Quality of Life (ADDQoL) [57], diabetes treatment satisfaction (DTS) [57], and EuroQol (EQ-5D) [59], and the short form of the state scale of the Spiegelberger State-Trait Anxiety Inventory [60]. All patients completed the consultation and relational empathy (CARE) measure in relation to the GP and practice nurse; participants in the intervention group also completed this measure in relation to the lifestyle facilitator [61]. Participants were also asked to complete six items at one year asking about their views on the diabetes service they had received in the last 12 months, including frequency of contact, information and advice received, and satisfaction with the service. Participants were also asked about the frequency of and views about self-monitoring of blood glucose using the Diabetes Glycaemic Education and Monitoring (DiGem) study questionnaire [62].

#### Beliefs about behaviour change, illness perceptions, and habit

A questionnaire, developed according to TPB guidelines [29,63], assessed selected cognitions about becoming more physically active, eating a lower fat diet, taking medication and smoking: intention, perceived behavioural control, and behavioural beliefs. Each construct was assessed with two items, measured on a 5-point Likert-type scale ranging from 'strongly disagree' to 'strongly agree'. Examples of perceived control items are: 'I am confident that I could be more physically active/eat a lower fat diet in the next 12 months, if I wanted to'; for intention: 'I intend to be more physically active/eat a lower fat diet in the next 12 months'; and for behavioural beliefs: 'If I was more physically active/did eat a lower fat diet in the next 12 months, it is likely that I would lose weight'. We used the consequences and treatment control subscales (11 items) of the Illness Perception Questionnaire-Revised (IPQ-R) [64,65]. The consequences scale assessed seriousness of diabetes and the impact of diabetes on various aspects of life. An example is: 'My diabetes is a serious condition'. The treatment control scale assessed beliefs and feelings associated with the management of diabetes. An example is: 'My treatment can control my diabetes'. Both scales were measured on 5-point Likert-type scales ranging from 'strongly disagree' to 'strongly agree'. Participants were also asked to complete a nine-item closed-response questionnaire covering basic knowledge of diabetes and its' management [66]. At one year, participants were asked to write down the most important change that they had made in their physical activity and dietary intake, and 12 items were used to assess to what extent these changes had become a habit [67].

#### Intervention evaluation

Participants in the intervention group completed 10 items at one year about their views on the frequency of contacts with the lifestyle facilitators, clarity of advice given, educational materials, and overall intervention. They also completed a skills questionnaire assessing their confidence in relation to using nine skills (e.g., goal setting, action planning, self-monitoring) on a 10-point scale ranging from 'not at all confident' to 'very confident', and use of these skills in the past 11 months in order to increase physical activity, eat a lower-fat diet, try to stop smoking (if applicable) and try to take medicines regularly as prescribed (if applicable) on a binary scale (yes/no).

#### Costs

Health service use in the three months prior to follow-up was quantified using an adapted version of the Health Services Research Unit Aberdeen questionnaire that enquires about the use of services (consultations with healthcare professionals and hospitalisations) and medications [68].

### Participant safety

Treatment algorithms were developed with advice from local diabetes specialists who also contributed to the initial and follow-up practice-based training sessions for primary care staff involved in diabetes care. The responsibility for prescribing and management decisions remained with the general practitioners. Classes of medication were only recommended within licensed indications. Before taking part in the treadmill test, participants completed a pre-test screening questionnaire incorporating the Rose angina questionnaire. If they answered positively to any of the questions, a short medical review was completed by a clinical member of the team. Blood pressure, current medications and ECG (if available) were also reviewed following strict protocols before a participant was allowed to begin the treadmill test.

### Statistical analysis

The additional benefit of the facilitator-led behaviour change intervention on objective measures of physical activity level, medication adherence, and change from baseline in plasma vitamin C and smoking status will be assessed using an intention to treat analysis comparing the intervention and comparison groups, using linear regression for continuous outcomes and a comparison of proportions for binary outcomes. Continuous outcomes will be analysed with adjustment for the baseline value of the outcome, where this has been measured, in order to improve precision. Those participants missing the baseline value will remain included in the analysis by using the missing indicator method, which is valid for pre-randomisation measures in trials [69]. Due to the large number of stratifier categories, there will be no adjustment for stratifiers in order to ensure that estimation of intervention effects is stable. The modelled ten-year cardiovascular risk will be log transformed before analysis and computed for those with and without a history of previous CVD events. The primary intention to treat analysis will be supported by per protocol analyses of primary outcomes, where the per protocol population is defined to be those participants attending the introductory and initial three core intervention sessions. The impact of missing primary outcome data will be examined within sensitivity analyses using the multiple imputation method of Rubin [70]. This will involve fitting an imputation model from the observed data, from which missing data will be imputed under two opposing scenarios. In the first scenario it is assumed that the same full intervention-minus-comparison effect applies amongst participants with missing data as has been observed amongst their counterparts with observed data. In the second scenario it is assumed that the intervention-minus-comparison effect is zero for participants with missing data. Ten complete datasets will be imputed, as recommended, to incorporate the uncertainty in the residuals and parameter estimates of the imputation model. Subgroup analyses will be confined to the comparison of intervention effects on primary outcomes by the route of diagnosis (screen-detected versus already-diagnosed).

The perspective for cost analysis will be the health service and will include recent use of services (GP/hospital appointments & admissions) and prescribed medications in the previous 3 months. The costs of intensive treatment with and without the facilitator-led behaviour change intervention will be compared with unit change in health utility. In addition, costs at one-year and future costs derived from existing data [71] will be compared with risk of death and cardiovascular events, with appropriate sensitivity analysis.

Cohort analyses will be reported separately and will test causal pathways between behavioural determinants, behaviour change and consequences of behaviour change.

### Sample size

The trial was designed within the main *ADDITION-Cambridge *trial. It was anticipated that 500 patients would participate in the *ADDITION-Plus *trial and that 200 individuals per group would be available with follow-up data. With these numbers, there is 80% power at the 5% level of significance level to detect the following relevant differences between arms in primary outcomes:

Physical activity:

A 0.017 kJ/kg/min difference in PAEE. This is based on a standard deviation of 0.058 at baseline in the *ProActive *trial dataset [72] with mean 0.078. A 0.017 absolute difference represents a 22% relative difference in mean energy expenditure between groups.

Diet:

A 5.5 umol/l difference in plasma vitamin C (control group mean 53, SD 19, EPIC study [73]). Adjustment for baseline level in the analysis allows a finer difference of 4.0 umol/l to be detected between arms (EPIC test-rest correlation of 0.67).

Medication adherence:

A 10% absolute difference in the proportion of low adherers to hypoglycaemic medications (control group 18%, Supported Adherence to Medication pilot study [51]).

Smoking:

A 9.5% absolute difference in the proportion smoking (control group 17.9%).

### Discussion

The *ADDITION-Plus *trial is designed to establish the medium-term effectiveness and cost-effectiveness of a facilitator-led intervention tailored to support change in multiple behaviours among intensively-treated individuals with recently diagnosed diabetes in primary care. Key features of this trial include (i) a carefully characterised target group and intervention; (ii) an intervention based on theory and evidence from psychology to support change in key behaviours affecting CVD risk - physical activity, dietary intake, medication adherence and smoking; (iii) quality assured delivery enabled by training, ongoing supervision and protocols; (iv) objective measurements of behaviours as well as self-report of behaviours, functional status and well-being, and; (v) an examination of the psychological, socio-demographic and clinical predictors of behaviour change as well as the clinical consequences of behaviour change in the cohort.

Results will inform policy recommendations concerning the introduction of health trainer initiatives out-with the primary-secondary care team to assist in early management of patients with type 2 diabetes. They will inform both the education of patients and practitioners, the quality assurance of behaviour change programmes, and will improve understanding of the association between changes in health behaviours and health outcomes.

## Competing interests

The authors declare that they have no competing interest.

## Authors' contributions

SJG, NJW, SS and ALK are the principal investigators for the *ADDITION-Plus *trial. AT is the trial statistician, WH is intervention programme co-ordinator, KMW is the trial co-ordinator, JG and FW are trial assistants, SB is an intervention facilitator, IH is a Clinical Psychologist, TF helped with data management and analysis, and SB and KW are part of the Physical Activity Technical team. RKS, SJG, WH, and ALK drafted the manuscript. All authors read and approved the final manuscript. SJG is the paper guarantor.

## Pre-publication history

The pre-publication history for this paper can be accessed here:

http://www.biomedcentral.com/1471-2458/11/211/prepub

## References

[B1] IDF The Diabetes Atlas, Fourth Edition 2009 IDF. Brussels

[B2] Diabetes UK Diabetes UK Impact Report 2008 Diabetes UK. London

[B3] Diabetes UK Diabetes in the UK 2004: A report from Diabetes UK 2004 Diabetes UK

[B4] Nothwehr F Stump T Health-promoting behaviors among adults with type 2 diabetes: findings from the Health and Retirement Study Prev Med 2000 30 5 407 414 10.1006/pmed.2000.0658 10845750

[B5] Glasgow RE Fisher EB Anderson BJ LaGreca A Marrero D Johnson SB Rubin RR Cox DJ Behavioral science in diabetes. Contributions and opportunities Diabetes care 1999 22 5 832 843 10.2337/diacare.22.5.832 10332691

[B6] Ismail K Winkley K Rabe-Hesketh S Systematic review and meta-analysis of randomised controlled trials of psychological interventions to improve glycaemic control in patients with type 2 diabetes Lancet 2004 363 9421 1589 1597 10.1016/S0140-6736(04)16202-8 15145632

[B7] Shojania KG Ranji SR McDonald KM Grimshaw JM Sundaram V Rushakoff RJ Owens DK Effects of quality improvement strategies for type 2 diabetes on glycemic control: a meta-regression analysis Jama 2006 296 4 427 440 10.1001/jama.296.4.427 16868301

[B8] Sperl-Hillen JM O'Connor PJ Factors driving diabetes care improvement in a large medical group: ten years of progress The American journal of managed care 2005 11 5 S177 185 16111440

[B9] Lindstrom J Ilanne-Parikka P Peltonen M Aunola S Eriksson JG Hemio K Hamalainen H Harkonen P Keinanen-Kiukaanniemi S Laakso M Sustained reduction in the incidence of type 2 diabetes by lifestyle intervention: follow-up of the Finnish Diabetes Prevention Study Lancet 2006 368 9548 1673 1679 10.1016/S0140-6736(06)69701-8 17098085

[B10] Knowler WC Barrett-Connor E Fowler SE Hamman RF Lachin JM Walker EA Nathan DM Reduction in the incidence of type 2 diabetes with lifestyle intervention or metformin N Engl J Med 2002 346 6 393 403 10.1056/NEJMoa012512 11832527 PMC1370926

[B11] Tuomilehto J Lindstrom J Eriksson JG Valle TT Hamalainen H Ilanne-Parikka P Keinanen-Kiukaanniemi S Laakso M Louheranta A Rastas M Prevention of type 2 diabetes mellitus by changes in lifestyle among subjects with impaired glucose tolerance N Engl J Med 2001 344 18 1343 1350 10.1056/NEJM200105033441801 11333990

[B12] Griffin S Diabetes care in general practice: meta-analysis of randomised control trials BMJ (Clinical research ed 1998 317 7155 390 396 9694757 10.1136/bmj.317.7155.390 PMC28634

[B13] Olivarius NF Beck-Nielsen H Andreasen AH Horder M Pedersen PA Randomised controlled trial of structured personal care of type 2 diabetes mellitus BMJ (Clinical research ed 2001 323 7319 970 975 10.1136/bmj.323.7319.970 11679387 PMC59690

[B14] Gaede P Lund-Andersen H Parving HH Pedersen O Effect of a multifactorial intervention on mortality in type 2 diabetes N Engl J Med 2008 358 6 580 591 10.1056/NEJMoa0706245 18256393

[B15] Wing RR Long-term effects of a lifestyle intervention on weight and cardiovascular risk factors in individuals with type 2 diabetes mellitus: four-year results of the Look AHEAD trial Archives of internal medicine 2010 170 17 1566 1575 10.1001/archinternmed.2010.334 20876408 PMC3084497

[B16] British Psychological Society Health Psychology Team Improving health: changing behaviour - NHS health trainer handbook 2008 DOH

[B17] Norris SL Nichols PJ Caspersen CJ Glasgow RE Engelgau MM Jack L Isham G Snyder SR Carande-Kulis VG Garfield S The effectiveness of disease and case management for people with diabetes. A systematic review Am J Prev Med 2002 22 4 Suppl 15 38 10.1016/S0749-3797(02)00423-3 11985933

[B18] Norris SL Engelgau MM Narayan KM Effectiveness of self-management training in type 2 diabetes: a systematic review of randomized controlled trials Diabetes care 2001 24 3 561 587 10.2337/diacare.24.3.561 11289485

[B19] Norris SL Zhang X Avenell A Gregg E Bowman B Serdula M Brown TJ Schmid CH Lau J Long-term effectiveness of lifestyle and behavioral weight loss interventions in adults with type 2 diabetes: a meta-analysis Am J Med 2004 117 10 762 774 10.1016/j.amjmed.2004.05.024 15541326

[B20] Goldstein MG Whitlock EP DePue J Multiple behavioral risk factor interventions in primary care. Summary of research evidence Am J Prev Med 2004 27 2 61 79 10.1016/j.amepre.2004.04.023 15275675

[B21] Padgett D Mumford E Hynes M Carter R Meta-analysis of the effects of educational and psychosocial interventions on management of diabetes mellitus J Clin Epidemiol 1988 41 10 1007 1030 10.1016/0895-4356(88)90040-6 3193136

[B22] Griffin S Kinmonth AL Skinner C Kelly J Educational and psychosocial interventions for adults with diabetes 1999 London: British Diabetic Association

[B23] Gaede P Vedel P Larsen N Jensen GV Parving HH Pedersen O Multifactorial intervention and cardiovascular disease in patients with type 2 diabetes N Engl J Med 2003 348 5 383 393 10.1056/NEJMoa021778 12556541

[B24] Kinmonth AL Griffin S Wareham NJ Implications of the United Kingdom Prospective Diabetes Study for general practice care of type 2 diabetes Br J Gen Pract 1999 49 446 692 694 10756608 PMC1313494

[B25] Mallion JM Dutrey-Dupagne C Vaur L Genes N Renault M Elkik F Baguet P Boutelant S Benefits of electronic pillboxes in evaluating treatment compliance of patients with mild to moderate hypertension J Hypertens 1996 14 1 137 144 12013487

[B26] Donnan PT Brennan GM MacDonald TM Morris AD For the DARTS/MEMO Collaboration UoD Population-based adherence to prescribed medication in type 2 diabetes: a cause for concern Diabet Med 2000 17 1 1 10.1046/j.1464-5491.2000.00251.x

[B27] Rothman AJ Baldwin A Hertel A Vohs K, Baumeister R Self-regulation and behaviour change: Disentangling behavioral initiation and behavioral maintenance The handbook of self-regulation 2004 New York: Guildford Press 130 148

[B28] Ellis SE Speroff T Dittus RS Brown A Pichert JW Elasy TA Diabetes patient education: a meta-analysis and meta-regression Patient Educ Couns 2004 52 1 97 105 10.1016/S0738-3991(03)00016-8 14729296

[B29] Ajzen I The Theory of Planned Behavior Organizational Behavior and Human Decision Processes 1991 50 179 211 10.1016/0749-5978(91)90020-T

[B30] Gollwitzer PM Brandstatter V Implementation intentions and effective goal pursuit Journal of Personality and Social Psychology 1997 73 186 199 10.1037/0022-3514.73.1.186 11708569

[B31] Lauritzen T Griffin S Borch-Johnsen K Wareham NJ Wolffenbuttel BH Rutten G The ADDITION study: proposed trial of the cost-effectiveness of an intensive multifactorial intervention on morbidity and mortality among people with Type 2 diabetes detected by screening Int J Obes Relat Metab Disord 2000 24 3 S6 11 10.1038/sj/ijo/0801420 11063279

[B32] Echouffo-Tcheugui JB Simmons RK Williams KM Barling RS Prevost AT Kinmonth AL Wareham NJ Griffin SJ The ADDITION-Cambridge trial protocol: a cluster -- randomised controlled trial of screening for type 2 diabetes and intensive treatment for screen-detected patients BMC Public Health 2009 9 136 10.1186/1471-2458-9-136 19435491 PMC2698850

[B33] Coleman R Stevens R Holman R The Oxford Risk Engine: A Cardiovascular Risk Calculator for Individuals with or without Type 2 Diabetes American Diabetic Association: 2007 2007 Chicago, USA A170 Diabetes

[B34] Horne R Weinman J Myers L, Midence K Predicting treatment adherence: an overview of theoretical models Adherence to treatment in medical conditions 1998 London Harwood Academic 25 50

[B35] Tight blood pressure control and risk of macrovascular and microvascular complications in type 2 diabetes: UKPDS 38 UK Prospective Diabetes Study Group Bmj 1998 317 7160 703 713 9732337 PMC28659

[B36] Intensive blood-glucose control with sulphonylureas or insulin compared with conventional treatment and risk of complications in patients with type 2 diabetes (UKPDS 33) UK Prospective Diabetes Study (UKPDS) Group Lancet 1998 352 9131 837 853 10.1016/S0140-6736(98)07019-6 9742976

[B37] Effect of intensive blood-glucose control with metformin on complications in overweight patients with type 2 diabetes (UKPDS 34) UK Prospective Diabetes Study (UKPDS) Group Lancet 1998 352 9131 854 865 10.1016/S0140-6736(98)07037-8 9742977

[B38] Pyorala K Pedersen TR Kjekshus J Faergeman O Olsson AG Thorgeirsson G Cholesterol lowering with simvastatin improves prognosis of diabetic patients with coronary heart disease A subgroup analysis of the Scandinavian Simvastatin Survival Study (4S) Diabetes Care 1997 20 4 614 620 10.2337/diacare.20.4.614 9096989

[B39] Effects of ramipril on cardiovascular and microvascular outcomes in people with diabetes mellitus: results of the HOPE study and MICRO-HOPE substudy Heart Outcomes Prevention Evaluation Study Investigators Lancet 2000 355 9200 253 259 10.1016/S0140-6736(99)12323-7 10675071

[B40] Collins R Armitage J Parish S Sleigh P Peto R MRC/BHF Heart Protection Study of cholesterol-lowering with simvastatin in 5963 people with diabetes: a randomised placebo-controlled trial Lancet 2003 361 9374 2005 2016 10.1016/S0140-6736(03)13636-7 12814710

[B41] Leventhal H Brissette I Leventhal EA Cameron LD, Leventhal H The common-sense model of self-regulation of health and illness The Self-Regulation of Health and Illness Behaviour 2003 London: Routledge 42 65

[B42] Diabetes for beginners- Type2 http://www.diabetes.org.uk http://www.diabetes.org.uk

[B43] Carver CS Scheier MF Wyer RS Themes and issues in the self-regulation of behavior Perspectives on behavioral self-regulation Advances in social cognition, Volume XII 1999 London: Lawrence Erlbaum Associates

[B44] Kazdin AE Behavior Modification in Applied Settings 2001 Belmont, CA: Wadsworth

[B45] Marlatt GA Gordon JR Relapse prevention: Maintenance strategies in the treatment of addictive behaviors 1985 New York: Guildford Press

[B46] Brage S Brage N Franks PW Ekelund U Wareham NJ Reliability and validity of the combined heart rate and movement sensor Actiheart Eur J Clin Nutr 2005 59 4 561 570 10.1038/sj.ejcn.1602118 15714212

[B47] Brage S Ekelund U Brage N Hennings MA Froberg K Franks PW Wareham NJ Hierarchy of individual calibration levels for heart rate and accelerometry to measure physical activity J Appl Physiol 2007 103 2 682 692 10.1152/japplphysiol.00092.2006 17463305

[B48] Tanaka H Monahan KD Seals DR Age-predicted maximal heart rate revisited Journal of the American College of Cardiology 2001 37 1 153 156 10.1016/S0735-1097(00)01054-8 11153730

[B49] Stegle O Fallert SV MacKay DJ Brage S Gaussian process robust regression for noisy heart rate data IEEE transactions on bio-medical engineering 2008 55 9 2143 2151 10.1109/TBME.2008.923118 18713683

[B50] Brage S Brage N Franks PW Ekelund U Wong MY Andersen LB Froberg K Wareham NJ Branched equation modeling of simultaneous accelerometry and heart rate monitoring improves estimate of directly measured physical activity energy expenditure J Appl Physiol 2004 96 1 343 351 10.1152/japplphysiol.00703.2003 12972441

[B51] Farmer AJ Prevost AT Hardeman W Craven A Sutton S Griffin SJ Kinmonth AL Protocol for SAMS (Support and Advice for Medication Study): a randomised controlled trial of an intervention to support patients with type 2 diabetes with adherence to medication BMC family practice 2008 9 20 10.1186/1471-2296-9-20 18405345 PMC2364626

[B52] Wareham NJ Rennie KL The assessment of physical activity in individuals and populations: why try to be more precise about how physical activity is assessed? Int J Obes Relat Metab Disord 1998 22 2 S30 38 9778094

[B53] Craig CL Marshall AL Sjostrom M Bauman AE Booth ML Ainsworth BE Pratt M Ekelund U Yngve A Sallis JF International physical activity questionnaire: 12-country reliability and validity Medicine and science in sports and exercise 2003 35 8 1381 1395 10.1249/01.MSS.0000078924.61453.FB 12900694

[B54] Bingham SA Gill C Welch A Cassidy A Runswick SA Oakes S Lubin R Thurnham DI Key TJ Roe L Validation of dietary assessment methods in the UK arm of EPIC using weighed records, and 24-hour urinary nitrogen and potassium and serum vitamin C and carotenoids as biomarkers International journal of epidemiology 1997 26 1 S137 151 10.1093/ije/26.suppl_1.S137 9126542

[B55] Rose GA The diagnosis of ischaemic heart pain and intermittent claudication in field surveys Bulletin of the World Health Organization 1962 27 645 658 13974778 PMC2555832

[B56] Feldman EL Stevens MJ Thomas PK Brown MB Canal N Greene DA A practical two-step quantitative clinical and electrophysiological assessment for the diagnosis and staging of diabetic neuropathy Diabetes Care 1994 17 11 1281 1289 10.2337/diacare.17.11.1281 7821168

[B57] Bradley C Handbook of psychology and diabetes: a guide to psychological measurement in diabetes research and practice 1994 Harwood Academic Press

[B58] Ware J Snow KK Kosinski M Gandek B SF-36 Health Survey Manual & Interpretation Guide 1993 Boston, Massachusetts: Nimrod press

[B59] Kind P Dolan P Gudex C Williams A Variations in population health status: results from a United Kingdom national questionnaire survey Bmj 1998 316 7133 736 741 9529408 10.1136/bmj.316.7133.736 PMC28477

[B60] Marteau TM Bekker H The development of a six-item short-form of the state scale of the Spielberger State-Trait Anxiety Inventory (STAI) Br J Clin Psychol 1992 31 3 301 306 1393159 10.1111/j.2044-8260.1992.tb00997.x

[B61] Mercer SW Maxwell M Heaney D Watt GC The consultation and relational empathy (CARE) measure: development and preliminary validation and reliability of an empathy-based consultation process measure Family practice 2004 21 6 699 705 10.1093/fampra/cmh621 15528286

[B62] Farmer A Wade A French DP Goyder E Kinmonth AL Neil A The DiGEM trial protocol--a randomised controlled trial to determine the effect on glycaemic control of different strategies of blood glucose self-monitoring in people with type 2 diabetes [ISRCTN47464659] BMC family practice 2005 6 25 10.1186/1471-2296-6-25 15960852 PMC1185530

[B63] Conner M Sparks P Conner M, Norman P Theory of planned behaviour and health behaviour Predicting health behaviour 2005 Open University Press 171 222

[B64] Leventhal L Benyamino Y Brownlee S Diefenbach M Leventhal EL Patrick-Miller L Robitaille C Petrie KJ, Weinman J Illness representations: theoretical foundations Perceptions of health and illness 1997 Amsterdam: Harwood Academic Publisher 19 45

[B65] Moss-Morris R Weinman J Petrie KJ Horne R Cameron LD Buick D The revised illness perception questionnaire (IPQ-R) Psychology & Health 2002 17 1 16

[B66] Kinmonth AL Woodcock A Griffin S Spiegal N Campbell MJ Randomised controlled trial of patient centred care of diabetes in general practice: impact on current wellbeing and future disease risk. The Diabetes Care From Diagnosis Research Team BMJ (Clinical research ed 1998 317 7167 1202 1208 9794859 10.1136/bmj.317.7167.1202 PMC28704

[B67] Verplanken B Orbell S Reflections on past behavior: A self-report index of habit strength Journal of Applied Pscyhology 2003 33 1313 1330 10.1111/j.1559-1816.2003.tb01951.x

[B68] Knapp M Beecham J Reduced list costings: examination of an informed short cut in mental health research Health economics 1993 2 4 313 322 10.1002/hec.4730020404 8142993

[B69] White IR Thompson SG Adjusting for partially missing baseline measurements in randomized trials Stat Med 2005 24 7 993 1007 10.1002/sim.1981 15570623

[B70] Schafer JL Multiple imputation: a primer Statistical methods in medical research 1999 8 1 3 15 10.1191/096228099671525676 10347857

[B71] Currie CJ Williams DR Peters JR Patterns of in and out-patient activity for diabetes: a district survey Diabet Med 1996 13 3 273 280 10.1002/(SICI)1096-9136(199603)13:3<273::AID-DIA57>3.0.CO;2-S 8689850

[B72] Williams K Prevost AT Griffin S Hardeman W Hollingworth W Spiegelhalter D Sutton S Ekelund U Wareham N Kinmonth AL The ProActive trial protocol - a randomised controlled trial of the efficacy of a family-based, domiciliary intervention programme to increase physical activity among individuals at high risk of diabetes [ISRCTN61323766 BMC Public Health 2004 4 1 48 10.1186/1471-2458-4-48 15491494 PMC526256

[B73] Bingham SA Welch AA McTaggart A Mulligan AA Runswick SA Luben R Oakes S Khaw KT Wareham N Day NE Nutritional methods in the European Prospective Investigation of Cancer in Norfolk Public Health Nutr 2001 4 3 847 858 10.1079/PHN2000102 11415493

[B74] Ware JE SK Kosinski M Gandek B SF-36 Health Survey. Manual and Interpretation Guide 1993 Boston, MA, New England Medical Centre, The Health Institute

